# Propolis Affects *Pseudomonas aeruginosa* Growth, Biofilm Formation, eDNA Release and Phenazine Production: Potential Involvement of Polyphenols

**DOI:** 10.3390/microorganisms8020243

**Published:** 2020-02-12

**Authors:** Aida Meto, Bruna Colombari, Agron Meto, Giorgia Boaretto, Diego Pinetti, Lucia Marchetti, Stefania Benvenuti, Federica Pellati, Elisabetta Blasi

**Affiliations:** 1 School of Doctorate in Clinical and Experimental Medicine, University of Modena and Reggio Emilia, Via G. Campi 287-103, 41125 Modena, Italy; aida.meto@unimore.it (A.M.); lucia.marchetti@unimore.it (L.M.); 2 Department of Surgery, Medicine, Dentistry and Morphological Sciences with interest in Transplant, Oncology and Regenerative Medicine, University of Modena and Reggio Emilia, Via G. Campi 287, 41125 Modena, Italy; bruna.colombari@unimore.it; 3 Department of Therapy, Faculty of Dental Medicine, University of Aldent, Rr. e Dibres 235, 1000 Tirana, Albania; agronmeto@yahoo.com; 4 School of Specialization in Microbiology and Virology, University of Modena and Reggio Emilia, Via G. Campi 287, 41125 Modena, Italy; giorgia.boaretto@virgilio.it; 5 Centro Interdipartimentale Grandi Strumenti, University of Modena and Reggio Emilia, via Campi 213/A, 41125 Modena, Italy; diego.pinetti@unimore.it; 6 Department of Life Sciences, University of Modena and Reggio Emilia, Via G. Campi 103, 41125 Modena, Italy; stefania.benvenuti@unimore.it

**Keywords:** *P. aeruginosa*, virulence factors, phenazines, eDNA, MIC, antimicrobial, antibiofilm, propolis, polyphenols

## Abstract

*Pseudomonas aeruginosa* (*P. aeruginosa*) is an opportunistic pathogen responsible for a wide range of clinical conditions, from mild infections to life-threatening nosocomial biofilm-associated diseases, which are particularly severe in susceptible individuals. The aim of this *in vitro* study was to assess the effects of an Albanian propolis on several virulence-related factors of *P. aeruginosa*, such as growth ability, biofilm formation, extracellular DNA (eDNA) release and phenazine production. To this end, propolis was processed using three different solvents and the extracted polyphenolic compounds were identified by means of high performance liquid chromatography coupled to electrospray ionization mass spectrometry (HPLC-ESI-MS) analysis. As assessed by a bioluminescence-based assay, among the three propolis extracts, the ethanol (EtOH) extract was the most effective in inhibiting both microbial growth and biofilm formation, followed by propylene glycol (PG) and polyethylene glycol 400 (PEG 400) propolis extracts. Furthermore, *Pseudomonas* exposure to propolis EtOH extract caused a decrease in eDNA release and phenazine production. Finally, caffeic acid phenethyl ester (CAPE) and quercetin decreased upon propolis EtOH extract exposure to bacteria. Overall, our data add new insights on the anti-microbial properties of a natural compound, such as propolis against *P. aeruginosa*. The potential implications of these findings will be discussed.

## 1. Introduction

In nature, many microbial species use a cell-to-cell signaling system, named quorum sensing (QS), to form biofilms on both biotic and abiotic surfaces. Microorganisms embedded in a biofilm acquire resistance to drugs and detergents and make host defenses less efficient [1,2,3]. Microbial biofilms consist of sessile cells, embedded in a self-produced matrix of polysaccharides, proteins, lipids and extracellular DNA (eDNA) [4,5]. *Pseudomonas aeruginosa* (*P. aeruginosa*), a Gram-negative, aerobic (and at times facultative anaerobic), encapsulated, rod-shaped bacterium, is an opportunistic pathogen responsible for nosocomial infections, which can be particularly severe and life threatening in susceptible individuals [6]. *P. aeruginosa* is responsible also for oral infections in patients with clinical conditions, such as apical periodontitis, pulp necrosis, pulpitis or mandibular/maxillary alveolitis [7]. Indeed, the onset and development of infections are mostly related to the well-established ability of *P. aeruginosa* to produce biofilm, either onto biotic or abiotic surfaces.

Several virulence factors of *P. aeruginosa*, including biofilm formation, pyoverdine and pyocyanin (PYO) production as well as eDNA release, are regulated by QS. In particular, eDNA release occurs through mechanisms involving the autoinducer N-acyl-l-homoserine lactones (AHL) and the *Pseudomonas* quinolone signaling (PQS) molecules. QS-independent mechanisms, via flagella and type IV pili, have also been documented as contributing to eDNA release [8]. PYO derives by the precursor phenazine-1-carboxylic acid (PCA), which is then converted in phenazine-1-carboxamide (PCN) and 1-hydroxy-phenazine (1-OH-PHZ) [9]; it is a virulence factor known to contribute to eDNA release by its interaction with molecular oxygen to form reactive oxygen species (ROS), such as hydrogen peroxide (H_2_O_2_), which cause cell lysis. In turn, the released eDNA promotes *Pseudomonas* adhesion and increases biofilm thickness and biomass content [10]. Recently, a rapid and easy-to-perform *in vitro* model has been described for real-time monitoring of *P. aeruginosa* growth and biofilm formation onto endotracheal tubes using a bioluminescent strain [11].

Traditional treatments of infectious diseases have broad-range efficacy. However, the frequency of microbial genetic mutations is drastically enhanced, resulting in an increased incidence of antibiotic resistance. Therefore, there is an increasing need to find other molecules, such as natural compounds, capable of killing or inhibiting microbial growth likely without promoting resistance mechanisms. In this context, propolis (bee-glue) is a well-known mixture of resinous and balsamic substances, that, collected by honey-bees from tree and herb buds, are transformed with the help of salivary secretions into a peculiar product. This natural-resinous hive substance has been considered as an official drug by London’s pharmacopoeia since the 17th century. The name propolis, derived from the ancient Greek, “pro” and “polis”, meaning “before or in defense of the city”. Indeed, propolis is used for beehive defense, as well as to close hive holes and cracks, in order to avoid contact with harmful agents, such as other insects, fungi, bacteria, etc. In Europe, propolis became very popular between the 17th and 20th centuries [12,13]. Its chemical composition and biological properties vary according to the geographical area and climate characteristics [14].

The main components of propolis are flavonoids, phenolic acids, terpenes, aromatic acids and other molecules, most of which are lipophilic and, therefore, easily dissolved in organic solvents, such as ethanol [15,16]. The biological activity of propolis is mostly linked to flavonoids and phenolic acids [17]. To date, propolis is considered as a potent antiseptic substance, successfully used in several clinical settings, without concomitant toxic/deleterious effects [12]. In particular, propolis has gained attention in different dentistry fields [17]; some clinical studies have demonstrated its preventive effect against dental caries [18] as well as its therapeutic benefits on oral surgery and periodontology [19], oral mucositis [20], plaque inhibition [21], pulp capping [22,23] or its efficacy as an endodontic irrigant [24]. Ethanol extract of propolis [25] has been demonstrated to possess also anti-inflammatory and regenerative effects, and this observation is confirmed by experiments in vivo of teeth pulpotomy in piglets [22]. Other important biological properties of propolis, ranging from antimicrobial [16], antibiofilm [26], anticancer [27], antioxidant [16,28], anti-inflammatory activities to wound-healing promotion [29] have been described; all these properties are attributed to its high polyphenols content.

The aim of this study was to assess *in vitro* the anti-*P. aeruginosa* activity of an Albanian propolis with respect to some virulence factors, such as biofilm formation, eDNA release and phenazine production. In addition, by high-performance liquid chromatography (HPLC) coupled with mass spectrometry (MS), the profile of propolis extracts was characterized, focusing on some specific polyphenols before and after exposure to *P. aeruginosa* cells.

## 2. Materials and Methods

### 2.1. Microbial Strain

The bioluminescent *P. aeruginosa* strain (P1242) (BLI-*Pseudomonas*) was maintained and handled as previously described [11]. This strain had been engineered to express the luciferase gene and luciferase substrate under the control of a constitutive P1 integron promoter. Thus, live cells constitutively produced a detectable bioluminescent signal [30]. Bacteria from −80 °C glycerol stocks were initially seeded onto Tryptic Soy Agar (TSA) (OXOID, Milan, Italy) plates and incubated overnight at 37 °C; then, isolated colonies were collected, added to 10 mL of Tryptic Soy Broth (TSB) (OXOID, Milan, Italy) and allowed to grow overnight at 37 °C with gentle shaking. Bacterial concentrations were then measured by the McFarland standard curve and appropriate dilutions prepared, according to the protocol.

### 2.2. Propolis

Crude propolis, produced by *Apis mellifera* L., was collected on June 2018, in north Albania (Puka, Thethi and Velë forest areas), far from asphalted urban or interurban roads. The woodland vegetation in this area, mainly consists of *Picea abies* L., *Populus alba* L., *Aesculus hippocastanum* L., *Castanea sativa* Miller, *Arbutus unedo* L., *Quercus pubescens* Wild., *Prunus dulcis* (Mill.) D.A. Webb, *Prunus avium* L., *Helianthus annuus* L., *Cucumis sativus* L., *Ulmus* L., *Thymus vulgaris* L., *Cornus mas* L., *Salvia officinalis* L., *Acacia penninervis* DC, *Salix* L., *Cytisus scoparius* L. & Link, *Trifolium* L. and *Carpinus* L. The crude propolis appeared as a mild amorphous mass, with an aromatic balsamic smell, in dark yellow to light brown color, with a bitter and burning taste numbing the oral mucosa. It was delivered at room temperature (RT) in the dark and kept at −80 °C until the extraction procedure.

### 2.3. Chemicals and Solvents

HPLC-grade acetonitrile (ACN), formic acid (HCOOH), analytical grade absolute ethanol (EtOH), propylene glycol (PG), polyethylene glycol 400 (PEG 400) and methanol (MeOH) were purchased from Sigma (Milan, Italy). Water (H_2_O) was purified using a Milli-Q Plus185 system from Millipore (Milford, MA, USA).

### 2.4. Extraction of Phenolic Compounds from Crude Propolis

One gram of an Albanian frozen propolis (kept at −80 °C) was grinded in a mortar and reduced to uniform particle size powder. The extraction was carried out by dynamic maceration with 10 mL of solvent (i.e., EtOH, PG and PEG 400) under the dark for 24 h, at RT. The extracts were centrifuged for 5 min at 4000 rpm. The supernatant solutions were filtered in a vacuum into a 10 mL volumetric flask and the solvents were added to the final volume. The extraction procedure was repeated twice for each solvent tested.

### 2.5. Spectrophotometric Analysis of Total Phenolics

The total phenolic content was determined by using the Folin-Ciocâlteu colorimetric assay with some modifications [31]. A solution of gallic acid in water, at different concentration (2–20 μg/mL), was used as the reference. The total phenolic content was assessed by using 50 μL of each extract, previously diluted 1:1 with H_2_O. Fifty μL of pure solvent (EtOH, PG and PEG 400) were used as the blank, respectively. Then, 500 μL of the Folin-Ciocâlteu reagent and 1 mL of sodium carbonate (Na_2_CO_3_) saturated solution were added. The solution was then adjusted to the final volume of 10 mL with H_2_O. Afterwards, the solutions were incubated at RT in the dark and, after 2 h, the absorbance was evaluated at 760 nm wavelength. The phenolic content was determined from the equation of the regression curve and expressed as mg of gallic acid equivalents for mL of propolis extract (mg GAE/mL).

### 2.6. Sample Preparation for HPLC Analysis

An aliquot of 200 μL of each propolis extract was properly diluted with 1 mL of EtOH in a volumetric flask, filtered through a 0.45 μm PTFE filter into a HPLC vial and injected in the HPLC system. All sample preparations were carried out in duplicate.

### 2.7. HPLC Analysis of Phenolics in Propolis Extracts

Chromatography was performed using an Agilent Technologies (Waldbronn, Germany) modular model 1100 system, consisting of a vacuum degasser, a quaternary pump, an autosampler, a thermostated column compartment and a diode array detector (DAD). The chromatograms were recorded using an Agilent ChemStation for LC and LC-MS systems (Rev. B.01.03).

The analysis was carried out on an Ascentis C_18_ column (250 × 4.6 mm I.D., 5 μm, Supelco, Bellefonte, PA, USA). The mobile phase was composed by (A) 0.1% HCOOH in H_2_O and (B) ACN. The gradient elution was modified as follows: 0–3 min 25% B, 3–10 min linear gradient from 25% to 30% B, 10–40 min from 30% to 40% B, 40–60 min from 40% to 60% B, 60–80 min from 60% to 90% B, 80–92 min 90% B. The post-running time was 5 min. The flow rate was 1.2 mL/min. The column temperature was set at 30 °C. The sample injection volume was 5 μL. The UV/DAD acquisitions were at 265 nm (for chrysin and galangin), 290 nm (for cinnamic acid, pinocembrin and pinobanksin), 320 nm (for caffeic acid, p-coumaric acid and ferulic acid), 338 nm (for apigenin and luteolin) and 370 nm (for quercetin, isorhamnetin and kaempferol).

HPLC coupled with electrospray ionization mass spectrometry (ESI-MS) analyses were performed using an Agilent Technologies modular 1200 system, equipped with a vacuum degasser, a binary pump, an autosampler, a thermostatted column compartment and a 6310 A ion trap mass analyzer with an ESI ion source. The HPLC column and the applied chromatographic conditions were the same as reported for the HPLC-DAD system. The flow-rate was split 6:1 before the ESI source. For ESI-MS^2^, the parameters were set as follows: the capillary voltage was 3.5 kV, the nebulizer (N_2_) pressure was 20 psi, the drying gas (N_2_) temperature was 350 °C, the drying gas flow was 9 L/min and the skimmer voltage was 40 V. Data were acquired by Agilent 6300 Series Ion Trap LC/MS system software (version 6.2). The mass analyzer was used in the full-scan positive and negative ion modes in the *m*/*z* range 100–1000. MS^2^ spectra were automatically performed with helium as the collision gas by using the SmartFrag function.

### 2.8. Minimal Inhibitory Concentration (MIC) Assay

The MIC assay was performed by the microbroth dilution method according to the Clinical and Laboratory Standards Institute/National Committee for Clinical Laboratory Standard (CLSI/NCCLS M7-A6) [32]. According to the experimental protocol, each propolis extract was tested at final dilutions, ranging from 500 µg GAE/mL to 1.9 µg GAE/mL. In parallel, each solvent (at the corresponding dilutions) and gentamicin (2 mg/mL) were included as negative and positive controls, respectively. A bacterial cell suspension (5 × 10^5^ cells/mL in Mueller Hinton plus 2% sucrose, obtained from overnight cultures) was seeded (100 μL/well) in a 96 U-bottom microtiter-plate; then, the bacterial cells were added with medium (100 µL/well) or treated with scalar doses of propolis extracts or their respective solvent (100 μL/well). Therefore, the plate was incubated at 37 °C for 24 h. The MIC of each extract was defined as the lowest concentration that inhibited visible *Pseudomonas* growth and in which the relative concentration of the solvent showed the minimal toxicity.

### 2.9. Assessment of Propolis Effects on Microbial Growth and Early Biofilm Formation

BLI-*Pseudomonas* is known to produce biofilm, as detailed elsewhere [11]. In order to monitor the total microbial growth under different experimental conditions, 100 µL of overnight cultures of BLI-*Pseudomonas* (5 × 10^5^/mL) in TSB plus 2% sucrose were seeded in a 96 black well-plate, containing 100 μL of TSB 2% sucrose (untreated) or propolis extract (treated) or solvent (control). The plates were then incubated at 35 °C for 16 h, into the Fluoroskan reader and the bioluminescence was detected at every hour. The values, collected in real time as a bioluminescence signal and expressed as relative luminescence units (RLU)/s, indicated the total microbial growth; based on an internal reference curve, such values could be converted in colony forming units (CFU)/mL.

In order to measure biofilm production by BLI-*Pseudomonas*, the samples were incubated for 16 h, washed twice with phosphate buffered saline (PBS) at RT to remove the planktonic cells and then the bioluminescence signal was measured. Such values were referred to the amounts of early biofilm formed onto plate surfaces, in treated or untreated samples.

### 2.10. Assessment of Phenazines and Propolis Polyphenols in Cell-Free Supernatants

In order to evaluate the amount of phenazines and to determine the levels of propolis polyphenols in supernatants from *Pseudomonas* exposed or not to propolis, a suitable HPLC-ESI-MS analysis was performed. To do this, all supernatants were filtered by Amicon Ultra-0.5 10 K centrifugal filter devices and diluted 1:5 (*v/v*) with 5% MeOH—0.2% HCOOH in H_2_O. The HPLC-ESI-MS instrument used was an UltiMate 3000 system, consisting of an online degasser, a binary pump HPG 3400RS, a well plate autosampler WPS 3000RS and a thermostatted column compartment TCC 3000RS coupled to a Q Exactive hybrid quadrupole–orbitrap mass analyzer via a HESI-II heated electrospray ion source (Thermo Scientific). Chromatographic separation of a 5 μL sample injection was performed on a Zorbax SB-C_18_ RRHT (50 × 2.1 mm I.D., 1.8 μm) column (Agilent) at 25 °C and a 0.3 mL/min flow rate. A linear gradient elution scheme was used with mobile phase components, being 0.1% HCOOH in H_2_O (A) and MeOH (B). The gradient started at 2% B, which was maintained for 0.5 min, then raised up to 42% B for 30 min and up again to 95% B for 4 min. The column was kept at 95% B for 4.4 min; then, the starting conditions were restored in 0.1 min and maintained for 11 min pending a successive injection. Electrospray ionization was operated in positive ion mode, using N_2_ as the sheath gas (40 arbitrary units), auxiliary gas (290 °C, 30 arbitrary units) and sweep gas (two arbitrary units). The sprayer voltage was kept at 3.5 kV and the transfer capillary temperature was set at 320 °C. The Q-Exactive was operated in Full MS/dd-MS^2^ mode. The full MS scan range was set from *m*/*z* 150 to 1000 at 70,000 full width at half maximum (FWHM) resolution (*m*/*z* 200). The automatic gain control (AGC) target was set at 1.0 × 10^6^ with a maximum injection time (IT) of 200 ms. Data-dependent MS^2^ (dd-MS^2^) acquisitions at 17,500 FWHM resolution (*m*/*z* 200) were triggered for the Top 3 precursor ions following each full MS scan. The intensity threshold for precursor ion selection was set to 1.0 × 10^5^ then dynamic exclusion was active for 20.0 s. AGC target and maximum IT for the MS^2^ experiments were set to 5.0 × 10^5^ and 80 ms.

### 2.11. Assessment of Propolis Effects on eDNA Release

For the analysis of eDNA, 100 µL of overnight cultures of BLI-*Pseudomonas* (5 × 10^5^/mL) in TSB plus 2% sucrose were seeded in 96 well-plate, containing 100 μL of medium (untreated) or propolis extract (treated) or solvent (control). The plates were then incubated at 35 °C for 16 h. The supernatants were collected and centrifuged twice at 14,000 rpm for 15 min and filtered by Amicon Ultra-0.5 10 K centrifugal filter devices in order to remove any remaining bacteria. To exclude residual viable bacteria, 50 μL of the supernatants were seeded onto TSA plates and incubated for 48 h at 37 °C under aerobic conditions; no bacterial CFU on TSA plates were ever observed. To quantify eDNA concentration in the cell-free supernatants, 100 μL of each sample were incubated with Propidium Iodide (PI) (1 μg/mL) for 15 min at 37 °C; then, the fluorescence emission was quantified by Fluoroskan reader (excitation/emission: 584/612).

### 2.12. Statistical Analysis

Quantitative variables were tested for normal distribution. Statistical differences between propolis treated and untreated (solvent) samples were analyzed according to Mann-Whitney test by using GraphPad prism 8. Values of *p* < 0.05 were considered significant.

## 3. Results

### 3.1. Total Phenolic Compounds in Propolis Extracts

Initially, the total polyphenol content of the three different propolis extracts (obtained by EtOH, PG and PEG 400) was determined by means of the Folin-Ciocâlteu colorimetric method. In particular, the PEG 400 extract showed the highest polyphenol content (5.8 ± 0.2 mg GAE/mL), while PG and EtOH extracts showed values of 4.8 ± 0.5 and 4.1 ± 0.4 mg GAE/mL, respectively.

### 3.2. HPLC Analysis of Polyphenols in Propolis Extracts

**Figure 1:**
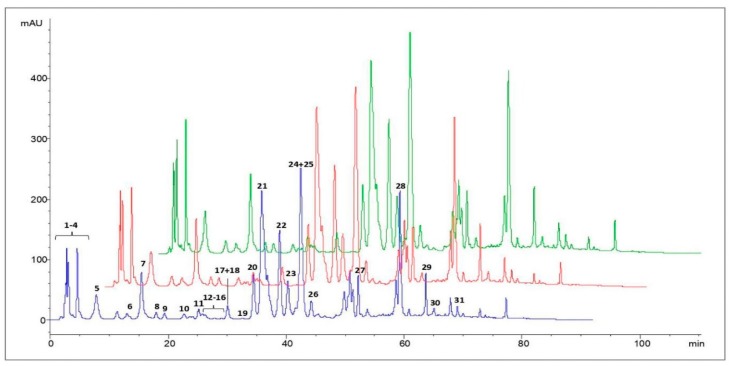
HPLC-UV/DAD chromatograms of EtOH (blue line), PG (red line) and PEG 400 (green line) propolis extracts. Data are from a representative experiment, out of two performed. For peak identification, see Table 1.

In order to investigate the presence of polyphenols on the three propolis extracts, a HPLC-UV/DAD analysis was performed and representative chromatograms are shown in Figure 1.

The compounds present in propolis extracts were identified by comparing the retention times of each peak with those of the standards, and by UV/Vis, MS and MS^2^ data [33]. The list of the polyphenols detected in propolis is shown in Table 1. The composition of the Albanian propolis extracts appeared qualitatively similar, irrespectively of the solvent used, likely because of the similar extraction properties of the solvents applied in this work.

### 3.3. Antibacterial Activity of Propolis

**Figure 2:**
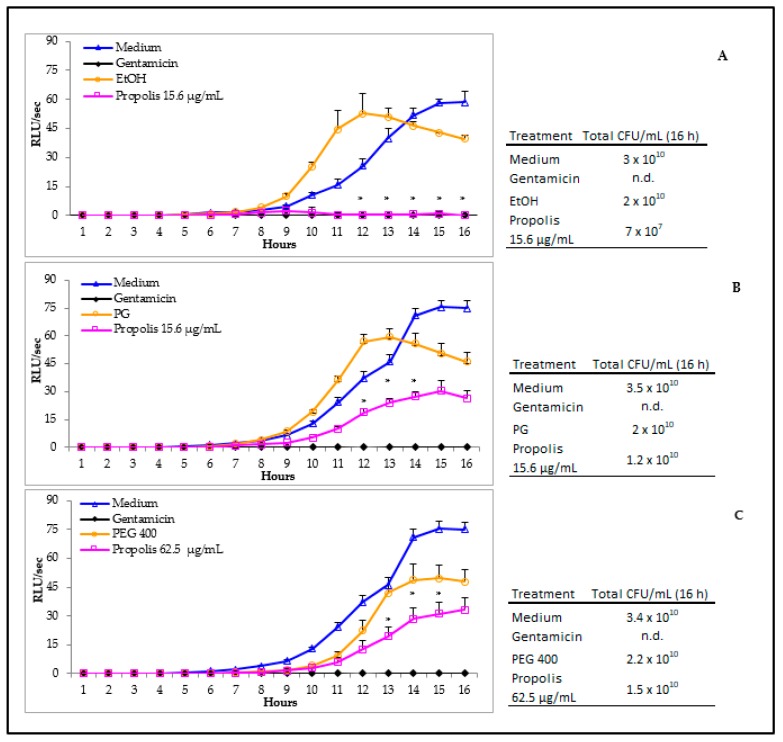
*P. aeruginosa* growth in the presence or absence of the three propolis extracts. The left panels show the total growth of *Pseudomonas* exposed to EtOH (**A**), PG (**B**) and PEG 400 (**C**) propolis extracts at their MICs, as measured kinetically by the BLI-based assay; in the right panels, the data are shown as total CFU/mL at 16 h. The respective solvents at the same dilutions were tested as negative controls; gentamicin (2 mg/mL) was used as the positive control. The results were expressed as mean ± SEM of the RLU/sec of 6–8 replicate samples obtained in two independent experiments. An internal calibration curve was used to convert to the RLU/s in total CFU/mL detectable at time 16 h. n.d.: not detectable. Statistical analysis was performed according to Mann Whitney test. * *p* < 0.05 propolis treated vs. solvent.

The antimicrobial activity of the three propolis extracts was evaluated *in vitro*, according to the standardized CLSI/NCCLS method [32]. In detail, nine different dilutions of each extract and their corresponding solvent dilutions were assessed by microbroth dilution. The MIC values obtained for both propolis EtOH and PG extracts were 15.6 μg/mL. Differently, the MIC obtained for propolis PEG 400 extract was as high as 62.5 μg/mL. Based on these results, a kinetic analysis of *Pseudomonas* growth upon exposure to each of the three propolis extracts was carried out, by means of a BLI-based assay known to provide direct and real time assessment of viable cells [11]. For each propolis extract, the MIC values and their corresponding diluted solvent or the medium alone were used (Figure 2). As depicted in Figure 2A, the RLU/s observed in the two controls (medium and EtOH) were similar, although an appreciable anticipation of the curve was observed with EtOH; in contrast, minimal or no RLU/s were ever detected in propolis extract treated samples. These differences were statistically significant, when comparing propolis treated- to solvent treated-*P. aeruginosa*, within the 12 to 16 h time frame. As shown in Figure 2B, in the medium and PG solvent controls, the RLU/sec had comparable time-related trends, with a slight anticipation by the latter. Propolis PG extract also significantly affected bacterial growth; in particular, a detectable signal occurred at 9 h reaching the highest value (30.47 RLU/s) at 15 h. Statistical significance was achieved at 12–14 h when comparing propolis extract with its solvent. Then, a slight decrease occurred in the luminescence signal down to 26.5 RLU/s at 16 h. When *P. aeruginosa* was grown in the presence or absence of PEG 400 propolis extract, a major toxicity of the solvent per se was evident (Figure 2C). In fact, all the RLU/s were consistently lower than the corresponding medium values. As for PG, the PEG 400 propolis extract did not completely affect bacterial growth. Statistical significance was reached within the 13–15 h time frame, when comparing propolis extract with solvent. When in parallel groups, gentamicin was used as the positive control, a complete inhibition of BLI-*Pseudomonas* growth was observed, as shown by the little or no luminescence signal detected (Panels A, B and C). Moreover, as depicted in Figure 2 (right panels), the conversion of the RLU in CFU/mL at 16 h allowed to underline that the most consistent inhibitory effects were indeed observed upon exposure to propolis EtOH extract (approximately 2.5 log decrease).

### 3.4. Propolis Effects on P. aeruginosa Early Biofilm Formation

**Figure 3:**
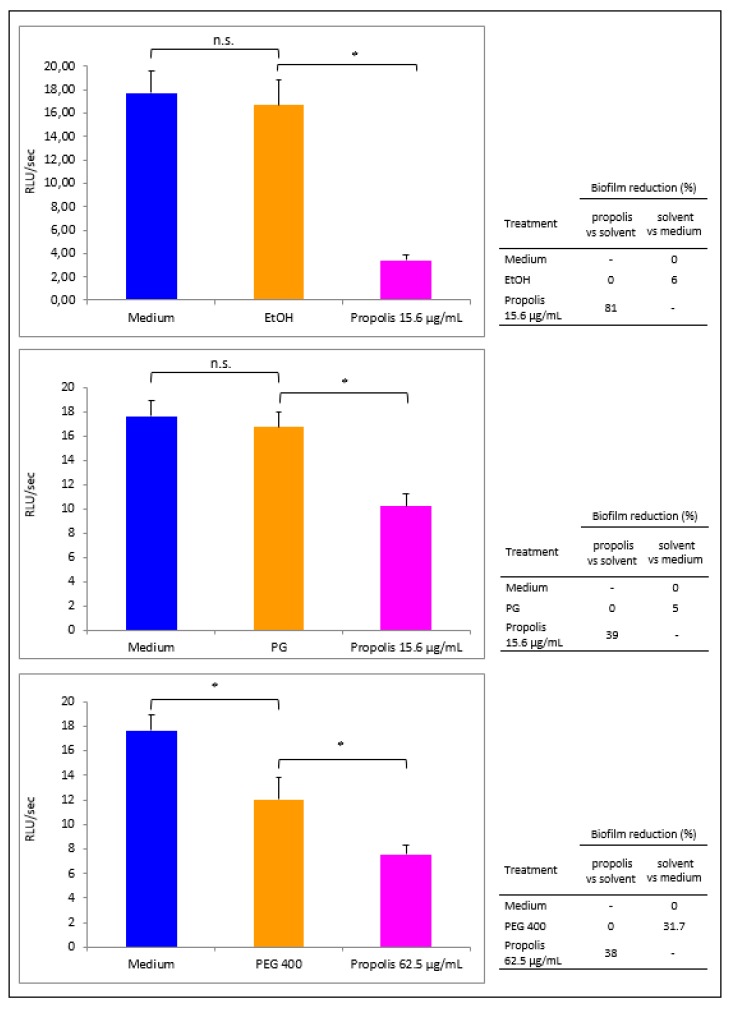
Propolis effects on early biofilm formation by *P. aeruginosa*. BLI-*Pseudomonas* cells were cultured for 16 h in medium or in the presence of EtOH (**A**), PG (**B**) and PEG 400 (**C**) propolis extracts, used at their MICs. Then, the wells were washed and the biofilm formation was quantified by a BLI assay. The luminescence values were plotted as mean ± SEM of 6–8 replicate samples of three independent experiments. Statistical analysis was performed according to Mann Whitney test. n.s.: not significant. * p < 0.05. The biofilm reduction (%) related to each condition is shown in the right inserts.

Bacterial cells, exposed or not to each propolis extract (used at its own MIC, i.e., 15.6 µg GAE/mL for propolis EtOH and PG extracts and 62.5 µg GAE/mL for propolis PEG 400 extract), were allowed to form a 16 h-old biofilm. Then, the wells were washed to remove the planktonic cells and the residual bioluminescent signal was evaluated to measure the early biofilm produced under the different experimental conditions (Figure 3). Propolis EtOH extract greatly prevented biofilm formation, as indicated by the low bioluminescent signal (panel A, pink column: 3.4 RLU/s). As shown in the right insert of Figure 3A, this drop corresponded to a significant inhibition (81%) in biofilm formation, when propolis extract was compared with the solvent. Differently, *Pseudomonas* cells exposed to EtOH alone produced a biofilm comparable to that developed in the presence of the medium alone (6% inhibition). When BLI-*Pseudomonas* was exposed to propolis PG extract, a still significant biofilm reduction (39%) was observed when propolis extract was compared with the solvent (right insert of Figure 3B), while the solvent per se had irrelevant effect (5% inhibition only). When PEG 400 was employed, an inhibitory effect by the solvent per se was observed (31.7%, with respect to medium); a further decrease occurred upon propolis PEG 400 extract treatment (38%, propolis vs. solvent) as shown in the right insert of Figure 3C.

### 3.5. Propolis Effects on Phenazines Release by P. aeruginosa

Phenazines are relevant virulence factors of *P. aeruginosa*. They are essential for adhesion and biofilm formation; they are also involved in oxidative stress, causing cell injury and death [9,10]. For this reason, the levels of three phenazines (PCA, PYO and 1-OH-PHZ) were assessed by HPLC-ESI-MS analysis, using *P. aeruginosa* cell-free supernatants from bacteria exposed to propolis EtOH extract, solvent or medium for 16 h. The results showed that propolis extract influenced the release of phenazines to a different extent, depending on the dose used. In particular, as shown in Table 2, the peak areas of PCA, PYO and 1-OH-PHZ decreased and this decrease (expressed as reduction percentage) ranged between 55% and 92.2%, depending on the propolis concentration. In all the cases, a relevant reduction was due to the solvent *per se*, when used at the condition corresponding to the highest propolis concentration.

### 3.6. Propolis Effects on eDNA release by P. aeruginosa

It is known that eDNA is a relevant component of *P. aeruginosa* biofilm, essential for the initial adhesion and stability of the sessile community [2,8]. In order to assess the capacity of *P. aeruginosa* to release eDNA, the bacteria were allowed to produce biofilm in the presence or absence of propolis extract for 16 h; then, cell-free supernatants were tested for the presence of eDNA, as detailed in the Materials and Methods. As shown in Table 3, eDNA release by *P. aeruginosa* was affected by propolis; in particular, the eDNA release decreased in a dose-dependent fashion, to 24.8% and 43.8% when using 15.6 and 31.2 µg/mL propolis, respectively. A partial reduction was also observed by the solvent *per se* (0.081 vs. 0.121 RFU; 33.1% decrease), when used at the condition corresponding to the propolis dose of 31.2 µg/mL. Moreover, a reduction of eDNA release was observed when comparing propolis vs. solvent; in this case, the RFU% reduction was 27.8 and 16.1, at 15.6 and 31.2 µg/mL respectively.

### 3.7. Polyphenol Content in Propolis Exposed or Not to P. aeruginosa

**Figure 4:**
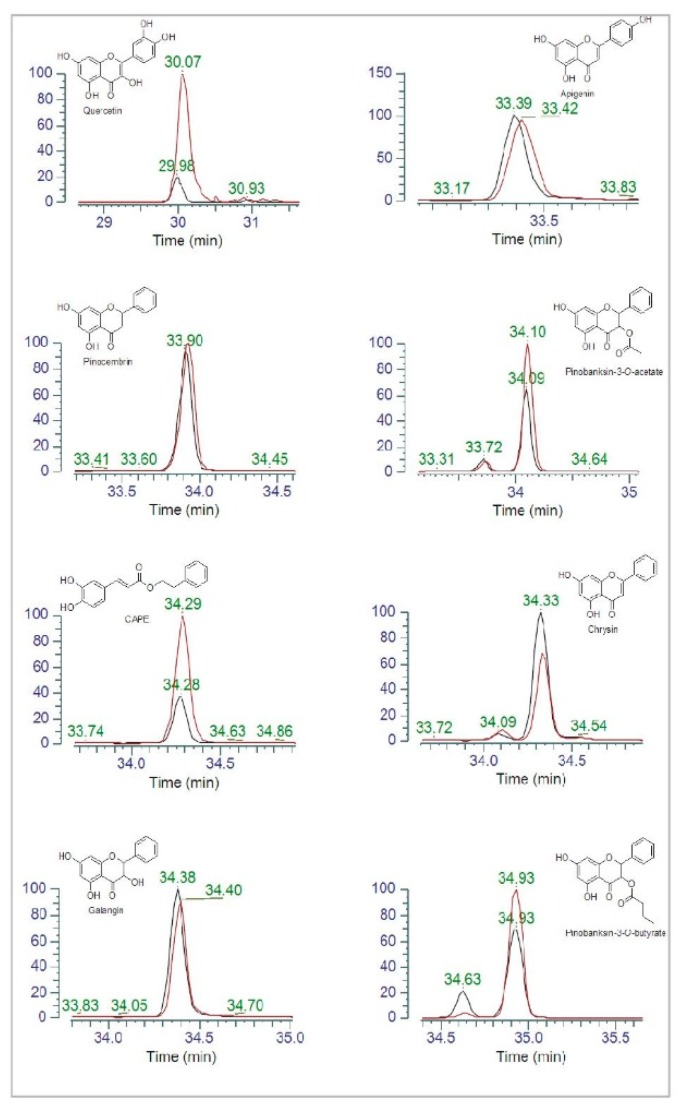
Overlapped peaks of eight polyphenols in propolis extract alone (red line) and upon exposure to *Pseudomonas* (black line). The chemical structure of each compound is also shown. Chromatograms were acquired in the negative ion mode. Data are shown according to the retention times (min).

Propolis is a complex mixture of components with a broad spectrum of activities, including antimicrobial, antioxidant, anti-inflammatory, anti-proliferative and anti-angiogenic effects [14,15,16]. A selected group of compounds, occurring in propolis and known for their biological activities, were analyzed by means of HPLC-ESI-MS. In particular, the peak areas observed in the propolis extract (15.6 µg/mL) exposed or not to *Pseudomonas* for 16 h were compared. The overlapped chromatographic peaks of eight polyphenols are shown in Figure 4. A reduction in the peak areas of quercetin and CAPE was observed upon propolis exposure to *P. aeruginosa* (black lines) with respect to the controls (propolis extract alone; red lines). A slight decrease was also observed for the pinobanksin-3-*O*-acetate and pinobanksin-3-*O*-butyrate peak areas, while that of chrysin moderately increased.

## 4. Discussion

Propolis is known to exert antimicrobial activity more efficaciously against Gram-positive than Gram-negative bacteria [34,35]; this is likely due to the peculiar structure of the latter as well as to their ability of producing a wide range of hydrolytic enzymes, which in turn likely break down the active compounds of propolis [34,35]. On these bases, our study focused on *Pseudomonas*, as a prototype of a hardly attackable pathogen, capable to express numerous virulence factors; its susceptibly to propolis has been evaluated in terms of variation in growth ability, biofilm formation, production of phenazines and eDNA release. In particular, an Albanian propolis, previously described for its therapeutic efficacy in dentistry settings [22], has been extracted using three different solvents, EtOH, PG and PEG 400, and then analyzed by HPLC-UV/DAD and HPLC-ESI-MS. The three profiles obtained, which happen to be comparable irrespectively of the solvent used, show that the most abundant flavonoids are chrysin, galangin, pinocembrin and pinobanksin (and its esters). Moreover, among phenolic acids, caffeic acid derivatives, such as CAPE, also occur in high amounts. Overall, the main components identified in the Albanian propolis closely recall those previously described for propolis samples of Italian origin and, more generically, of European origin [33]. Hereafter, the rough extracts have directly been tested (i.e., without solvent removal) against *Pseudomonas* to avoid loss of any volatile compounds, likely mediating antimicrobial activities [33].

When expressed in terms of MIC, the anti-*Pseudomonas* activity of each propolis extract varies from 15.6 μg/mL for EtOH and PG extracts to a four-fold higher value (62.5 μg/mL) for the PEG 400 extract. To better investigate the phenomenon, a highly sensitive bioluminescence-based model has been used [11], in order to real-time monitor both total microbial growth and viable cells organized as biofilm. Preliminary results, aimed at testing serial dilutions in the sub-MIC range, provided evidence for inconsistent inhibitory effects (data not shown), thus orienting the focus of our efforts on the MIC condition. As detailed in the Results section, relevant differences among the three extracts have been observed. In particular, the propolis EtOH extract happens to be the most efficient in decreasing the total microbial growth (the solvent *per se* showing no toxic effects). In contrast, the efficacy of the other two extracts is partially hampered by the toxicity of both PG and PEG 400 solvents. The anti-*Pseudomonas* effect of propolis EtOH extract, demonstrated by the persistently low BLI signal, is further highlighted when converting the bioluminescence values in CFU/mL at 16 h. Thus, propolis EtOH extract appears as the most interesting anti-*Pseudomonas* among the three extracts tested. Further analysis has revealed that EtOH propolis significantly reduces (81% decrease) the amounts of viable-metabolically active cells, capable of producing biofilm; in contrast, only a partial reduction (26%) of the total biofilm mass occurs under that same condition, as assessed by crystal-violet staining (data not shown), thus suggesting that the amounts of viable cells and, to a lesser extent, the polymeric extracellular matrix accumulation are indeed affected by propolis.

The BLI-based assay allowed us to demonstrate a significant reduction (39%), also when using propolis PG extract. Differently, the propolis PEG 400 extract inhibitory effects are somehow hampered by the action due to the solvent *per se* (31.7% inhibitory effect). Thus, by a highly sensitive BLI-based system, these findings provide novel evidence on the anti-*Pseudomonas* activity of propolis extracts, underlying that its efficacy also depends on the solvent used. Whether the anti-*Pseudomonas* effects of propolis are mainly due to a direct antibacterial activity or rather to a specific antibiofilm effect remains to be elucidated. Further experiments aimed at evaluating propolis effects on pre-formed *Pseudomonas* biofilm have revealed a consistent lack of activity independently on the propolis extract considered (data not shown), thus implying that propolis acts as an anti-*Pseudomonas* agent when the pathogen is in a planktonic stage, while an already structured sessile microbial community appears to be insensitive.

*P. aeruginosa* produces a large amount of water-soluble blue-green phenazine pigments, known to exert antimicrobial [36] and antifungal [37] activities. Phenazines control colony size, favor bacterial adhesion and increase thickness and biomass of *P. aeruginosa* biofilm [38,39]. Moreover, PYO, one of the most studied phenazines, interacts with molecular oxygen to form reactive oxygen species, like H_2_O_2_, which modify the redox balance. By our study, we expanded the knowledge on *Pseudomonas* as a phenazine producer, demonstrating not only PYO but also PCA and 1-OH-PHZ release. Moreover, a semiquantitative analysis, performed by comparing the peak areas of each compound under different conditions, indicated that phenazine production occurs to a similar extent in untreated cells (i.e., grown in medium) and in cells exposed to EtOH solvent. Differently, when comparing controls vs. propolis treated groups, a dose-dependent reduction in the peak areas is observed, implying that the overall release of such virulence factors is affected (always >50% decrease) in the presence of propolis. A further analysis, carried out by normalizing phenazine production with respect to the area described under the cell growth curve within the period of 16 h (i.e., total viable cells), indicated an increase of several fold in phenazine production upon propolis treatment (see Table S1). This apparent discrepancy suggests that the single cell secretory activity of the surviving/persisting cells is indeed increased, opening to the hypothesis of a microbial reaction to propolis-mediated insult. Further in-depth studies are needed to elucidate this point.

eDNA, which has been demonstrated to support biofilm stability, is abundantly released by *Pseudomonas* [5,8]. In our model, eDNA levels detected following propolis treatment are impaired in a dose-dependent manner. This decrease is probably related to the reduced number of cells detected at the end of the treatment. In any case, in line with other studies [10], the reduction of eDNA is shown to parallel with the decreased production of PYO.

It is well-known that propolis composition may vary as a function of the geographical area of production, botanical sources, season of collection, etc. [15]. Its therapeutic efficacy is mainly related to its antimicrobial, antioxidant, antiproliferative effects. The capacity of propolis to modulate the immune system has also been described [40,41,42]. As main propolis components, polyphenols have been investigated to understand the mechanisms involved in propolis-mediated effects [43]. It has been reported that the antibacterial mechanism of quercetin probably depends on the disruption of target membrane and inactivation of extracellular proteins by forming irreversible complexes [44]. In addition, quercetin and its derivatives may reduce the expression of some inflammatory genes. The effects of this compound have been described in the murine RAW264.7 macrophage cell line, in terms of oxigenase-1 protein production, transduction of nuclear factor NFkB, decrease in Nrfk2 gene expression and inactivation of miR-155 [45]. Caffeic acid and CAPE have a significant role in cellular cycle and cancer cell apoptosis; bacterial replication also seems to be affected [46,47]. Chrysin and its phosphate ester exert a strong inhibitory effect on Enterovirus EV71 [48]. Galangin significantly suppresses growth of vancomycin-intermediate *Staphylococcus aureus* (VISA) strain Mu50 [49]. Veloz et al. [50] have shown that pinocembrin and apigenin are able to modify the architecture of *S. mutans* biofilm, reducing its thickness; antimicrobial activity against *S. mutans* has also been documented [50]. In our *in vitro* model, BLI-*Pseudomonas* exposure to propolis has been shown to reduce the levels of several phenolic compounds, as shown by a decrease in their corresponding peak areas; in particular, quercetin and CAPE are consistently affected, while pinobanksin-3-O-acetate and pinobanksin-3-O-butyrate appear to slightly decrease when compared to the control (propolis alone). On the contrary, chrysin slightly increases in the supernatants of bacteria treated with propolis. Given the antimicrobial properties of quercetin and CAPE, we favor the idea that these compounds can directly interact with bacteria, thus explaining the decrease observed upon propolis exposure to *Pseudomonas*. A similar phenomenon also occurs when considering pinobanksin esters, known for their antioxidant effects [51]. Whether these compounds may be involved in the inhibition or modulation of oxidation reactions remains to be investigated. Taken together, these data provide an initial evidence that *Pseudomonas* affects several polyphenols present in propolis; further in-depth studies are warranted to better address this issue.

## 5. Conclusions

Our results strengthen the relevance of propolis as a natural antimicrobial product against *P. aeruginosa*, a Gram-negative opportunistic pathogen, known to be highly refractory to disinfectants, antibiotics and host defense mechanisms due to its multiple virulence-factors. By a highly sensitive luminescence-based model, here, we provide the first evidence that *Pseudomonas* exposure to propolis impairs its growth ability, production of biofilm and capacity to release molecules, such as phenazines and eDNA. Being these peculiarities closely related to *Pseudomonas* pathogenic potential, we may envisage propolis as a precious source of natural compounds for the development of new therapeutic strategies, particularly against biofilm-related infections. In this perspective, it will be interesting to evaluate the antibacterial activity of specific propolis components, widening the panel of both polyphenolics and microbial agents as well.

## Figures and Tables

**Table 1:** HPLC-UV/DAD, HPLC-ESI-MS and MS^2^ data obtained for the analysis of propolis constituents.

Peak Number	Compounds	UV λ_max_(nm)	[M + H]^+^	[M − H]^−^	MS^2^ Fragments (*m*/*z)*
**1**	Caffeic acid	298,324		179	135
**2**	*p*-Coumaric acid	298,310		163	119
**3**	Ferulic acid	298,324		193	149, 134
**4**	Isoferulic acid	296,321		193	149, 134
**5**	3,4-Dimethyl-caffeic acid (DMCA)	296,322		207	163, 133
**6**	Quercetin	256,372	303		285, 257, 229, 165, 153, 149
**7**	Pinobanksin-5-methyl-ether	288,318 sh	287		269, 241, 152, 91
**8**	Quercetin-3-methyl-ether	256,358	317		302, 165, 153, 137
**9**	Chrysin-5-methyl-ether	264,314	269		254, 167
**10**	Apigenin	267,338	271		253, 153, 119
**11**	Pinobanksin	291,330 sh	273		255, 227, 153
**12**	Isorhamnetin	255,372	317		302, 285, 177, 153
**13**	Luteolin-methyl-ether	266,350	301		286, 217
**14**	Quercetin-dimethyl-ether	254,356	331		316, 301, 299
**15**	Galangin-5-methyl-ether	260,302 sh, 352	285		270, 239, 167
**16**	Pinobanksin-5-methyl-ether-3-*O*-acetate	288,326	329		287, 241
**17**	Cinnamilidenacetic acid	312	175		157, 129
**18**	Quercetin-7-methyl-ether	256,372	317		302, 271, 243, 179, 167
**19**	Quercetin-dimethyl-ether	256,357	331		316, 299
**20**	Caffeic acid prenyl ester	298,326		247	179, 135
**21**	Chrysin	268,314 sh	255		209, 153, 129
**22**	Pinocembrin	290,330 sh	257		215, 153, 131, 103
**23**	Galangin	260,308 sh, 360	271		165, 153, 105
**24**	Caffeic acid phenylethyl ester (CAPE)	298,328		283	179, 135
**25**	Pinobanksin-3-*O*-acetate	294,332 sh	315		273, 255, 227, 153
**26**	Methoxy-chrysin	266,310 sh, 340 sh	285		270, 257, 242
**27**	Pinobanksin-3-*O*-propionate	292,330 sh	329		273, 255, 227, 153
**28**	Pinobanksin-3-*O*-butyrate *	268,310 sh	343		273, 255, 227, 153
**29**	Pinobanksin-3-*O*-pentanoate *	292,332 sh	357		273, 255, 227, 153
**30**	Pinobanksin-3-*O*-hexanoate *	282	371		273, 255, 227, 153
**31**	*p*-Methoxy cinnamic acid cinnamyl ester	278	295		149

Experimental conditions as in Section 2.7. * Or positional isomers. Data are from a representative experiment, out of two performed.

**Table 2:** Propolis effects on phenazines release by *P. aeruginosa*.

	Phenazines								
	PCA			PYO			1-OH-PHZ		
		Reduction (%)			Reduction (%)			Reduction (%)	
Treatment	Peak Area	Propolis vs. Medium	Propolis vs. Solvent	Peak Area	Propolis vs. Medium	Propolis vs. Solvent	Peak Area	Propolis vs. Medium	Propolis vs. Solvent
Medium	2.7 × 10^9^	0	-	5.1 × 10^9^	0	-	4.1 × 10^7^	0	-
EtOH	3 × 10^9^	-	0	5.3 × 10^9^	-	0	4.4 × 10^7^	-	0
Propolis 15.6 µg/mL	9.7 × 10^8^	64	68	2.3 × 10^9^	55	56.6	1.7 × 10^7^	58.5	61.4
EtOH	1.7 × 10^9^	-	0	3 × 10^9^	-	0	2.4 × 10^7^	-	0
Propolis 31.2 µg/mL	2.2 × 10^8^	92	87.1	5 × 10^8^	90.2	83.4	3.2 × 10^6^	92.2	86.7

The supernatants of BLI-*Pseudomonas* exposed to the medium, propolis EtOH extract or solvent for 16 h were collected and tested for phenazines levels by HPLC-ESI-MS analysis. The peak area values of the three phenazines (PCA, PYO and 1-OH-PHZ) in their specific chromatographic runs were used for semiquantitative evaluation. The percentage reduction was expressed with respect to the medium alone or to the solvent. The results shown are from a representative experiment out of two performed.

**Table 3:** Propolis effects on eDNA release by *P. aeruginosa*.

	eDNA		
Treatment	RFU	Reduction (%)	
		Propolis vs. Medium	Propolis vs. Solvent
Medium	0.121	0	-
EtOH	0.126	-	0
Propolis 15.6 µg/mL	0.091	24.8	27.8
EtOH	0.081	-	0
Propolis 31.2 µg/mL	0.068	43.8	16.1

The eDNA content was determined in 16 h cell-free supernatants from *P. aeruginosa,* exposed or not to propolis. PI was added before fluorescence reading, as detailed in the Materials and Methods. The results were expressed as mean fluorescence values (RFU) of triplicate samples. Standard deviations values < 5% were omitted. The reduction (%) was calculated with respect to the medium and the solvent. These values are from a representative experiment out of two performed.

## Supplementary Materials

The following are available online at https://www.mdpi.com/2076-2607/8/2/243/s1.
