# Supplementary material for: Propolis Affects Pseudomonas aeruginosa Growth, Biofilm Formation, eDNA Release and Phenazine Production: Potential Involvement of Polyphenols
Source: Microorganisms. 2020 Feb 12;8(2):243. doi: 10.3390/microorganisms8020243 (PMC7074903; doi:10.3390/microorganisms8020243)
Supplement: Supplementary file 1 [file microorganisms-08-00243-s001.pdf]

Phenazine production normalized according to the amounts of total viable cells

Treatment	Area under the growth curve	Phenazine production (fold increase)[a]		
		PCA	PYO	1-OH-PHZ
Medium	$1x10^{11}$	-	-	-
Propolis 15.6 µg/mL	$4x10^{9}$	8.8	11	10

BLI-*Pseudomonas* cells were cultured for 16 h in medium or in the presence of EtOH propolis extract (15.6 µg/mL). Then, the supernatants were harvested, filtered and analyzed by HPLC-ESI-MS to assess phenazine content. According to the BLI growth, kinetically recorded within the 16 h, the areas under each growth curve were calculated using the trapezoid rule method. Then, such areas (i.e. total viable cells) were used to normalize the amounts of phenazine detected in the supernatants.

[a] The indicated values represent the fold increase in phenazine production, considering the ratio between treated and untreated groups.
